# Meta-Learner-Based Approach for Detecting Attacks on Internet of Things Networks

**DOI:** 10.3390/s23198191

**Published:** 2023-09-30

**Authors:** Shaza Dawood Ahmed Rihan, Mohammed Anbar, Basim Ahmad Alabsi

**Affiliations:** 1 Applied College, Najran University, King Abdulaziz Street, Najran P.O. Box 1988, Saudi Arabia; sdrihan@nu.edu.sa (S.D.A.R.); baalabsi@nu.edu.sa (B.A.A.); 2 National Advanced IPv6 (NAv6) Centre, Universiti Sains Malaysia, Gelugor 11800, Malaysia

**Keywords:** Internet of Things, deep-learning models, Internet of Things attacks, meta-learning approach

## Abstract

The significant surge in Internet of Things (IoT) devices presents substantial challenges to network security. Hackers are afforded a larger attack surface to exploit as more devices become interconnected. Furthermore, the sheer volume of data these devices generate can overwhelm conventional security systems, compromising their detection capabilities. To address these challenges posed by the increasing number of interconnected IoT devices and the data overload they generate, this paper presents an approach based on meta-learning principles to identify attacks within IoT networks. The proposed approach constructs a meta-learner model by stacking the predictions of three Deep-Learning (DL) models: RNN, LSTM, and CNN. Subsequently, the identification by the meta-learner relies on various methods, namely Logistic Regression (LR), Multilayer Perceptron (MLP), Support Vector Machine (SVM), and Extreme Gradient Boosting (XGBoost). To assess the effectiveness of this approach, extensive evaluations are conducted using the IoT dataset from 2020. The XGBoost model showcased outstanding performance, achieving the highest accuracy (98.75%), precision (98.30%), F1-measure (98.53%), and AUC-ROC (98.75%). On the other hand, the SVM model exhibited the highest recall (98.90%), representing a slight improvement of 0.14% over the performance achieved by XGBoost.

## 1. Introduction

The Internet of Things (IoT) has emerged as a transformative force in modern technology, characterized by its exponential growth and pervasive influence. IoT has vastly grown in recent years, affecting nearly all areas of our daily existence. IoT devices have become integral to our world, from smart homes that intelligently manage temperature and lighting preferences to interconnected industrial systems streamlining manufacturing processes. This proliferation is not solely due to technological advances but also stems from the tangible advantages they offer individuals and industries. IoT devices bring unparalleled convenience into our daily routines, enabling remote control of household appliances, enhancing energy efficiency, and facilitating seamless access to information. This remarkable growth, coupled with the promise of elevated living standards, underscores the profound significance of IoT in our modern age [1].

In this interconnected era, the need for robust IoT attack detection is not just a security measure but an absolute necessity. IoT devices have woven themselves into the fabric of contemporary society, touching everything from smart homes to industrial machinery. Consequently, IoT networks have become enticing targets for malicious actors seeking to exploit vulnerabilities.

The repercussions of IoT attacks loom large, posing substantial threats to data security and critical infrastructure. Among these, data breaches are particularly concerning. IoT devices frequently collect and transmit sensitive personal and organizational data, from health records to financial information. Insufficient security measures can expose these data to unauthorized access, leading to identity theft, financial losses, and severe privacy breaches. Additionally, the interconnected nature of IoT networks means that one breached device can serve as a gateway for further infiltration, amplifying the risks.

Beyond data breaches, IoT attacks can disrupt vital infrastructure systems, potentially compromising industrial control systems, transportation networks, and healthcare devices. Disruptions can significantly affect public safety, economic stability, and essential services.

Given these formidable challenges, the importance of proactive and adaptable detection mechanisms cannot be overstated. Although effective in their own right, traditional intrusion detection systems (IDS) face considerable hurdles in IoT attack detection. These systems operate based on predefined rules and known attack signatures, making them ill-suited for IoT threats’ dynamic and rapidly evolving landscape [2]. IoT devices generate a large volume of data that can overwhelm traditional IDS, causing a high rate of false alarms.

In response to these challenges, meta-learning emerges as a compelling solution. Meta-learning represents a paradigm shift in cybersecurity, offering a dynamic and adaptive approach to threat detection. Meta-learners, trained to learn from past experiences and tasks, continually evolve their understanding of IoT attacks. This adaptability allows them to recognize new attack patterns and behaviors that may elude traditional IDS. By harnessing the learning capabilities of meta-learning, we can address the limitations of traditional IDS and stay one step ahead in the ever-evolving landscape of IoT security threats [3]. The contribution of this paper is as follows:

- Stacking DL models based on three DL models, recurrent neural Networks (RNN), Long Short-Term Memory Networks (LSTM), and Convolutional Neural Networks (CNN), are designed and developed.
- An approach based on meta-learners is proposed for detecting attacks on IoT attacks. This meta-learner approach is trained using the output of the stacked DL models.
- A thorough evaluation of different meta-learner models is conducted to assess the influence of the stacked DL models on the performance of the meta-learner. Furthermore, a comprehensive evaluation of DL models in detecting unseen IoT attacks.

The subsequent sections of the paper are structured as follows: In Section 2, we delve into the relevant prior research. Section 3 gives an overview of the research background. The intricate details of the approach are outlined in Section 4. Section 5 is dedicated to showcasing the outcomes of our experiments. We round off the paper by presenting conclusions and outlining potential future directions in Section 6.

## 2. Related Works

In their study, Zhang et al. [4] presented an alternative strategy involving multiple feature fusion and a uniform stacking ensemble to identify anomalies in network traffic. They crafted various features and trained multiple base classifiers with the same characteristics. The predictions from these base classifiers were amalgamated via a Random Forest (RF) meta-classifier to reach the ultimate decision.

In the work by Gao et al. [5], an adaptive learning-based ensemble approach was introduced to address the intricacies of intrusion datasets. This ensemble incorporated five distinct classifiers: decision tree, Random Forest (RF), k-nearest neighbors (kNN), and deep neural network (DNN), functioning as individual base learners. Decisions were made through majority voting, with varying weights assigned to the decisions of each classifier. This ensemble methodology was specifically employed for detecting intrusions within the NSL-KDD Test+ dataset.

In the research by Zhou et al. [6], a heterogeneous ensemble based on voting was crafted. They integrated the CFS-BA hierarchical feature extraction algorithm to enhance feature representation during preprocessing. Their proposed methodology integrated shallow algorithms, including Forest Penalizing Attributes, C4.5, and RF, on the extracted representation. An average voting technique was employed to consolidate the outcomes from the base classifiers.

In a separate study, Chalé et al. [7] introduced an intrusion detection framework rooted in meta-learning. This framework synergizes user input and data element attributes to determine the optimal algorithm for identifying cyberattacks. Subsequent experiments were conducted on the NSL-KDD dataset. The findings revealed that this framework alleviates the uncertainty associated with conventional trial-and-error algorithm selection techniques, consistently opting for the algorithm with superior classification performance.

Ahsan et al. [8] applied a stacked ensemble meta-learning approach using Dynamic Feature Selector (DFS), integrating various algorithms including CNN + LSTM, Bi-directional LSTM (BiLSTM), GRU, DT, and Random Forest. Their method dynamically selects features based on instance training results to improve prediction accuracy. Experimental findings showcased its efficacy on the NSL-KDD and UNSWNB15 datasets. For NSL-KDD, feature size was reduced from 123 to 50, elevating accuracy from 99.54% to 99.64%. In the case of UNSWNB15, accuracy increased from 90.98% to 92.46%, with the feature count shrinking from 196 to 47. The approach demonstrated enhanced accuracy and a remarkable reduction in feature requirements.

Olasehinde [9] pioneered meta-learning for intrusion detection solutions. The researcher introduced a novel intrusion detection method employing three meta-level algorithms within a stacked ensemble framework. This approach utilized Naive Bayes (NB) and Decision Tree (DT) to train the three meta-learning algorithms—MDT, MLR, and MMT. The evaluation was conducted using the UNSWNB15 test dataset for the foundational layer and the meta-stack models. The results underscored that the intrusion detection accuracy achieved by the three meta-learner models surpassed the highest accuracy of each respective original base model.

In 2020, Xu et al. [10] introduced a pioneering application of well-established meta-learning theory to few-shot intrusion detection systems (IDS). They presented a detection approach, FC-net, founded on a meta-learning framework. The algorithm is rooted in the principles of deep neural networks and is primarily structured with a feature extraction network and a comparison network. Experimental findings demonstrated the method’s versatility in intrusion detection, extending beyond specific attack types. Results from training and testing on datasets indicated that the proposed approach achieved an average detection rate of 98.88%. It also exhibited a capacity to effectively identify malicious samples in untrained datasets, with an average detection rate of 99.62% in select cases.

Alghanam et al. [11] introduced LS-PIO, an improved version of pigeon-inspired optimization (PIO), which integrates a local search algorithm to enhance the performance of a network intrusion detection system (NIDS) for IoT security. Their NIDS employs ensemble learning with multiple one-class classifiers and is assessed using benchmark datasets, including BoT-IoT, UNSW-NB15, NLS-KDD, and 99. The findings indicate that their approach surpasses other NIDS methods from contemporary literature.

Syed et al. [12] proposed a novel IoT intrusion detection framework for fog-cloud deployment. It involves distributed data processing, dataset segmentation based on attack class, and feature selection for time-series IoT data. Deep-learning techniques are employed for attack detection, particularly Recurrent Neural Networks (SimpleRNN and Bi-directional Long Short-Term Memory LSTM). Their evaluation of the BoT-IoT dataset demonstrates that feature selection significantly reduces data size by 90% while maintaining effective attack detection. Models trained on the reduced dataset exhibit higher recall rates than those using the full feature set without compromising class differentiation.

In another study [13], researchers presented an approach for detecting DoS attacks using deep machine-learning algorithms, incorporating the evaluation of RF, CNN, and MLP algorithms. They utilized hash chains as a threat model for IoT devices, offering a secure mechanism for storing and relocating device records.

Saba et al. [14] proposed a CNN-based approach for anomaly-based IDS tailored to IoT’s capabilities, enabling efficient examination of entire IoT traffic. Their model can detect potential intrusions and abnormal traffic behavior. They trained and tested the model using the NID Dataset and BoT-IoT datasets, achieving accuracy rates of 99.51% and 92.85%, respectively.

A mutual information (MI)–based anomaly detection technique for IoT attacks detection using deep neural networks (DNNs) is proposed by [15]. Using the IoT-Botnet 2020 dataset, various deep-learning models are compared and contrasted. These models include DNNs, CNN, RNN, and their variations, such as Gated Recurrent Units and LSTM. The experimental findings demonstrate the efficacy of the DNN-based NIDS model in comparison to the well-known deep-learning models, with an improvement in model accuracy of 0.57–2.6% and a reduction in FAR of 0.23–7.98%.

In [16] work, authors designed an efficient botnet detection model to enhance detection performance. The research improves the initial population generation strategy of the Dung Beetle Optimizer (DBO) by replacing the original random generation strategy with the centroid opposition-based learning strategy. The enhanced DBO is then applied to optimize Catboost parameters within the domain of IoT-Botnet detection. Real-world IoT traffic datasets are used in performance comparison experiments. The results of these experiments indicate that the proposed method outperforms other models in terms of accuracy and F1 score, affirming its effectiveness in the field. This literature review explores the development and evaluation of these botnet detection models in detail.

In [17] work, a framework for botnet detection is introduced, utilizing both machine-learning and deep-learning models. The study leverages the IoT-23 dataset and employs feature engineering to identify the key features crucial for botnet detection. Machine-learning algorithms, including SVM, Decision Tree, Random Forest, and Naive Bayes, are trained on the IoT-23 dataset using the selected features obtained through feature ranking. Although SVM demonstrates strong precision, it comes with a high time complexity. In contrast, Random Forest and Decision Tree models exhibit lower time complexity. To further enhance detection accuracy, two deep-learning models, CNN and GRU, are developed for botnet detection. GRU outperforms CNN, achieving an impressive accuracy rate of approximately 99.87%.

The authors in paper [18] proposed a collaborative machine-learning model for the early detection of IoT-Botnet based on multiple features, going beyond the use of full time-series data. The model utilizes specific data of the IoT-Botnet, such as system calls, network flow, and equipment resource appropriation. By incorporating these features, the proposed model aims to improve the early detection time and detection accuracy of IoT-Botnet attacks. Traditional malware detection methods typically rely on monitoring a single type of feature or data. Still, this paper’s collaborative machine-learning model combines multiple features using collaborative learning techniques. This approach allows for a more comprehensive analysis of IoT-Botnet behavior and enhances the effectiveness of early detection. The model achieves an accuracy of 99.37% on a dataset of 5023 IoT botnets and 3888 benign samples, demonstrating its effectiveness in detecting IoT-Botnet attacks.

The authors in paper [19] conducted a comparative study of deep-learning approaches for intrusion detection: deep discriminative and generative/unsupervised models. Specifically, the authors analyzed seven deep-learning approaches, including recurrent neural networks, deep neural networks, restricted Boltzmann machines, deep belief networks, convolutional neural networks, deep Boltzmann machines, and deep autoencoders. These machine-learning methods are compared using two new datasets, the CSE-CIC-IDS2018, and the BoT-IoT datasets, with three important performance indicators: false alarm rate, accuracy, and detection rate. Table 1 summarizes the studies on IoT-attacks detection.

In summary, researchers employ machine-learning techniques, including ensemble methods, deep-learning models, traditional classifiers, and meta-learning approaches. Among the deep-learning models commonly used and show impressive results are CNN, RNN, and LSTM. Therefore, this research utilized CNN, RNN, and LSTM to build the proposed meta-learner approach.

## 3. Background

IoT attacks and their implications are discussed in this section. Furthermore, a brief introduction to meta-learner is provided, as shown in Section 3.1 and Section 3.2, respectively.

### 3.1. IoT Attacks and Their Implications

Typical IoT attacks include distributed denial of service (DDoS) attacks where many compromised devices are used to overwhelm a target network or website with traffic, rendering it inaccessible [20,21,22]. Another common type is man-in-the-middle (MitM) attacks, where an attacker intercepts and alters the communication between IoT devices, potentially gaining unauthorized access to sensitive information or controlling the devices remotely. Additional attack types, including those related to IoT botnets, are listed in Table 2.

The potential consequences of IoT attacks on individuals and organizations can be severe. Individuals’ personal information and privacy may be compromised, leading to identity theft or financial loss. Additionally, IoT attacks on organizations can result in significant financial damage, disruption of operations, and loss of customer trust. Furthermore, the interconnected nature of IoT devices means that a successful attack on one device can potentially lead to a domino effect, compromising the entire network or system [23,24]. Robust detection systems are crucial to identify and mitigate IoT attacks promptly. Implementing advanced security measures such as encryption, authentication protocols, and regular software updates can help strengthen the overall security of IoT devices and networks. Additionally, educating individuals and organizations about the potential risks associated with IoT and promoting responsible usage can also contribute to minimizing the impact of attacks. The most prominent security challenges in IoT are listed in Table 3.

### 3.2. Meta-Learning

Meta-learning, often called “learning-to-learn”, debuted in the educational science community before its application in machine learning. Before its incorporation into machine learning, Maudsley [26] first used the term “meta-learning” in 1979. Presently, meta-learning stands as a significant research field within machine learning. Researchers have made notable strides in this area, particularly in harnessing meta-learning for tasks such as hyperparameter optimization, refining neural networks, and determining optimal network architectures. Model-based, metric-based, and optimization-based meta-learning approaches are the main camps in the current meta-learning research body [27,28]. Furthermore, novel meta-learning models have emerged in recent years. When considering insights from cybersecurity, these models can be broadly classified into two classes: online-learning-based methods and stacked ensemble-based methods.

In meta-learning, a “meta-learner” is trained on a collection of tasks, each comprising a task-specific dataset. These tasks can be viewed as learning experiences, and the meta-learner learns from them to acquire general knowledge or “meta-knowledge”. This meta-knowledge helps the model generalize better across different tasks and datasets, making it more adaptable and efficient in learning new concepts [28].

In IDS, meta-learning algorithms play a pivotal role in enhancing IDS capabilities by allowing the system to continuously adapt and improve its ability to identify and respond to emerging threats. These algorithms empower the IDS to anticipate attacks by analyzing vast data volumes, identifying trends, and drawing insightful conclusions. Machine-learning algorithms also reduce false positives by accurately distinguishing between normal network behavior and suspicious activities, yielding more efficient and effective threat identification [29].

A key advantage of employing meta-learning for IoT attack detection is its proficiency in learning from new and evolving attack patterns. This aspect is especially crucial in the dynamic landscape of IoT security, where conventional rule-based systems might struggle to keep pace. Moreover, meta-learning capitalizes on insights from past attacks to rapidly identify and counter potential threats, thus augmenting the overall effectiveness of security measures within the IoT ecosystem. Another significant differentiation lies in the extent of flexibility and adaptability [30]. Traditional ML models usually have a fixed structure and require retraining when confronted with new tasks or datasets. Conversely, meta-learning models possess the capacity to dynamically tailor their internal representations and weights to match various tasks. This adaptability is advantageous when data distributions shift over time or continuous learning is imperative.

## 4. Proposed Approach

This section explains an approach to detect attacks on IoT networks using a meta-learner approach. The relevance of meta-learning in IoT attack detection lies in its ability to tackle the challenges outlined in the IoT attacks section effectively. Meta-learning is particularly well-suited for IoT security due to its capacity to adapt and generalize across various attack scenarios and dynamically evolving threats.

In the IoT landscape, attacks can take on diverse forms and adapt rapidly, making it challenging to develop traditional, static detection models. Meta-learning offers a solution, emphasizing learning from different tasks and adapting to new, unseen instances. It enables IoT security systems to continually learn and evolve, becoming more adept at recognizing novel attack patterns and emerging threats without needing constant manual intervention [30].

**Figure 1:**
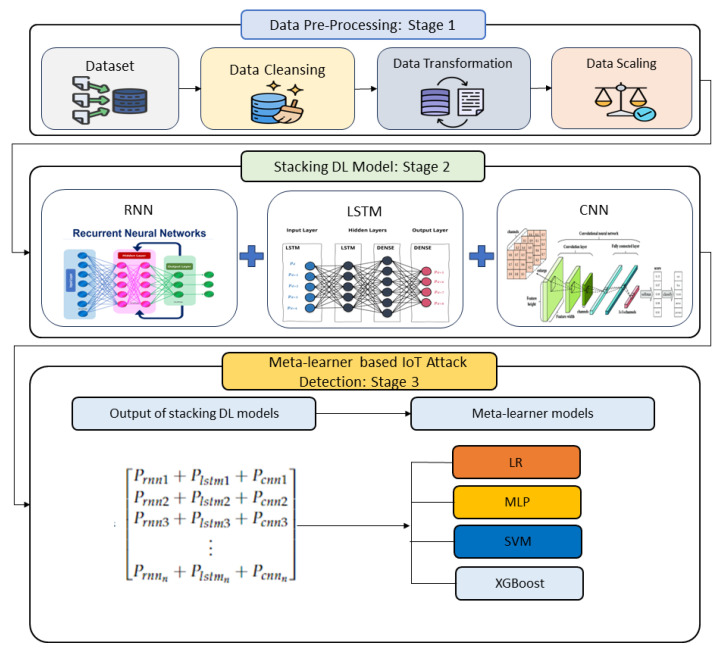
Main stages of the proposed approach.

Furthermore, meta-learning can leverage the vast data IoT devices generate to improve detection accuracy. By quickly adapting to changing attack tactics and leveraging these data, it enhances the IoT security framework’s ability to identify and respond to attacks in real time. The proposed approach consists of three stages, namely: (1) data preprocessing, (2) stacking DL models, and (3) a meta-learning model for detecting IoT attacks. Figure 1 shows the main stages of the proposed approach.

### 4.1. Data Preprocessing

Data preprocessing is pivotal across all Machine-Learning (ML) and Deep-Learning (DL) methods. Applied to the evaluation dataset, this stage centers on refining and converting raw data into a format conducive to analysis. It addresses missing values, removes outliers, and encodes categorical variables. By executing data preprocessing, the dataset’s integrity and dependability are augmented, thus fortifying the precision and effectiveness of ML/DL-based approaches during evaluation [31,32]. In this research, we applied normalization and data transformation and removed missing values from the dataset before feeding it to the DL models.

### 4.2. Stacking DL Models

This section elucidates the base classifiers employed in constructing the stacking prediction model. We utilized three deep-learning-based classifiers for this purpose, as explained in the subsequent sections.

#### 4.2.1. Base Model 1

The first base classifier is an RNN [33]; RNN belongs to a category of artificial intelligence neural networks considering the current input and their past input observations. This implies the presence of a secondary memory input. In this framework, the RNN’s decision at time t − 1 impacts its decision at time *t*. Consequently, the RNN processes input from two distinct sources, the current input and recent past data, collaborating to determine its response to new data. The primary distinguishing factor between RNNs and feed-forward neural networks is the presence of a feedback loop. Unfortunately, one of the drawbacks of RNNs is the vanishing gradient problem, which arises when the gradient becomes extremely small, hindering weight adjustments and preventing further training of the neural network.

The architecture used in this research is based on [34]. The prediction output of the RNN model is an n×2 array due to the final output layer having 2 units. Each unit corresponds to a different class (e.g., normal and attack). The SoftMax activation function converts the output values into probabilities for each class. Therefore, the output is a probability distribution over the two classes, with each entry in the array representing the predicted probability for that class. The RNN output probabilities array (RNN_probabilities) denotes as below:

$$\begin{array}{c} \mathrm{RNN\_probabilities}=\left[\begin{array}{cc} rnn_{label0} & rnn_{label1}\\ rnn_{label0} & rnn_{label1}\\ \vdots & \vdots \\ rnn_{labeln} & rnn_{labeln} \end{array}\right] \end{array}$$

#### 4.2.2. Base Model 2

The second base classifier is LSTM [35]; LSTM is a type of RNN proposed to combat the vanishing gradient issue seen in conventional RNNs. Unlike regular RNNs, which generate a new hidden state using the previous hidden state and the current input, LSTM not only does this but also considers old cell states. An LSTM cell typically comprises three gates: the input gate, the forget gate, and the output gate. These gates are calculated using the following equations:

(1) $$i_{t}=\sigma \;\left(W_{i}\;\left[\;h_{t-1},x_{t}\;\right]+b_{i}\right)$$

(2) $$f_{t}=\sigma \;\left(W_{f}\;\left[\;h_{t-1},x_{t}\;\right]+b_{f}\right)$$

(3) $$o_{t}=\sigma \;\left(W_{o}\;\left[\;h_{t-1},x_{t}\;\right]+b_{o}\right)$$

(4) $$h_{t}=o_{t}\;o\;tanh\;\left(c_{t}\right)$$

(5) $$c_{t}=f_{t}\;o\;c_{t-1}\;+\;i_{t}\;o\;{\tilde{c}}_{t}$$

(6) $${\tilde{c}}_{t}=tanh\;\left(w_{c}\;\left[h_{t-1},x_{t}\right]\;+\;b_{c}\right)$$

Equation (1) defines the input gate, while Equation (2) specifies the forget gate, and Equation (3) provides the formula for the output gate. The tanh activation function, which confines the output within the range of −1 and 1, can be substituted with alternative activation functions if desired. These three gates are responsible for modulating the input data and the memory from the previous time step to produce the output. The memory calculation is represented by Equation (4), which is derived by multiplying the output data from the current output gate with the cell state after passing through the tanh function. This memory captures the short-term memory component generated by the interplay between the output and the long-term memory. The cell state, which represents the long-term memory, is computed using Equation (5). It involves multiplying the cell state from the previous time step (modified by the forget gate) by the candidate state. Equation (6) outlines the calculation for the candidate state, which encapsulates the information intended to be stored in the cell state. The architecture of LSTM used in this research is based on [34]. The prediction output of the RNN model is an n×2 array due to the final output layer having 2 units. The LSTM output probabilities array (LSTM_probabilities) denotes as below:

$$\begin{array}{c} \mathrm{LSTM\_probabilities}=\left[\begin{array}{cc} lstm_{label0} & lstm_{label1}\\ lstm_{label0} & lstm_{label1}\\ \vdots & \vdots \\ lstm_{labeln} & lstm_{labeln} \end{array}\right] \end{array}$$

#### 4.2.3. Base Model 3

The third base classifier is a CNN [36]; CNNs have been widely used in various computer vision applications, including object detection, image classification, and facial recognition. Their ability to capture spatial and temporal patterns makes them well-suited for processing visual media and time-series data. CNNs use convolutional layers to learn and extract information autonomously from input, disregarding the requirement for human-engineered features. These convolutional layers use trainable filters to conduct element-wise multiplication and summing of the input data, yielding a feature map. The mathematical expression for this operation can be represented as follows:

(7) $$F\left(i,j\right)=\sigma \left(\sum_{m}\sum_{n}K\left(m,n\right)I\left(i+m,j+n\right)+b\right)$$

where *F* represents the output feature map, *I* represents the input data, *K* represents the filter set (or kernels), sigma represents the activation function, *b* represents the bias term, and (i,j) represent the spatial coordinates of the output feature map. To decrease the dimensionality of the data and provide a more manageable output after the features have been extracted, they are passed through a series of pooling layers. Finally, classification and regression tasks are performed on one or more fully linked layers. The CNN architecture used in this research is based on [37]. The prediction output of the CNN model is an n×2 array due to the final output layer having 2 units. The CNN output probabilities array (CNN_probabilities) denotes as below:

$$\begin{array}{c} \mathrm{CNN\_probabilities}=\left[\begin{array}{cc} cnn_{label0} & cnn_{label1}\\ cnn_{label0} & cnn_{label1}\\ \vdots & \vdots \\ cnn_{labeln} & cnn_{labeln} \end{array}\right] \end{array}$$

The RNN, LSTM, and CNN models are trained using the top ten best features reported in [37]. RNN_probabilities, LSTM_probabilities, and CNN_probabilities are input for the next stage to construct the stacking prediction model.

#### 4.2.4. Stacking Prediction Models

Stacking prediction models is an ensemble technique combining multiple individual predictive models to create a more powerful model. Stacking can often enhance predictive performance by leveraging the strengths of different models and mitigating individual model weaknesses [38,39,40].

Considering that the label data for both the training and testing datasets consists of one-dimensional arrays (n×1), where each index corresponds to either 1 (attack) or 0 (normal), and the input for this stage is an n×2 array (RNN_probabilities, LSTM_probabilities, and CNN_probabilities), we transform the n×2 array into an n×1 array by extracting the class with the highest probability for each row before proceeding to stack the three models as illustrated in Algorithm 1.
**Algorithm 1** Transforming and Stacking Predictions  1:Let n2 be the length of the input n2 array RNN_probabilities, LSTM_probabilities[i],andCNN_probabilities  2:Initialize an empty 1n array RNN_prob_1n of size n1  3:Initialize an empty array LSTM_prob_1n of size n1  4:Initialize an empty array CNN_prob_1n of size n1  5:**for** *i* from 1 to n2 **do**  6:   **for** *j* from 1 to n2[i].length **do**  7:     Let max_class be the index of the class with the highest probability in RNN_probabilities[i][j]  8:     Append max_class to RNN_prob_1n  9:     Let max_class be the index of the class with the highest probability in LSTM_probabilities[i]10:     Append max_class LSTM_prob_1n11:     Let max_class be the index of the class with the highest probability in CNN_probabilities[*i*]12:     Append max_class to CNN_prob_n113:   **end for**14:**end for**15:Stacking_input ← RNN_prob_1n, LSTM_prob_1n, CNN_prob_1n16:Stack_models_arr using Stacking_input as input

The Stack_models_arr is denoted as below:

$$\begin{array}{cc} Stack\_models\_arr=\left[\begin{array}{c} \mathrm{RNN\_prob}\\ P_{rnn1}\\ P_{rnn2}\\ P_{rnn3}\\ \vdots \\ n \end{array}\right]+\left[\begin{array}{c} \mathrm{LSTM\_prob}\\ P_{lstm1}\\ P_{lstm2}\\ P_{lstm3}\\ \vdots \\ n \end{array}\right]+\left[\begin{array}{c} \mathrm{CNN\_prob}\\ P_{cnn1}\\ P_{cnn2}\\ P_{cnn3}\\ \vdots \\ n \end{array}\right]\; & =\left[\begin{array}{c} P_{rnn1}+P_{lstm1}+P_{cnn1}\\ P_{rnn2}+P_{lstm2}+P_{cnn2}\\ P_{rnn3}+P_{lstm3}+P_{cnn3}\\ \vdots \\ P_{rnn_{n}}+P_{lstm_{n}}+P_{cnn_{n}} \end{array}\right] \end{array}$$

The output of the Stack_models_arr process results in a structured n×3 array. This array is designed and constructed to serve as the input data for training the meta-learner classifier. Each row in this n×3 array corresponds to an instance or data point, and the three columns represent the outputs or predictions generated by the three underlying models, namely RNN, LSTM, and CNN. This aggregation process is instrumental in providing a comprehensive and diversified set of features derived from multiple models, which is then used to train the meta-learner classifier. By combining the predictions from these different models, the meta-learner can gain a more holistic understanding of the data, potentially improving its ability to make accurate and robust predictions.

### 4.3. Meta-Learning-Based Model for Detecting IoT Attack

In this stage, the selection of the meta-learning model is determined through experimental analysis of multiple classifiers trained using Stack_models_arr. These classifiers include Logistic Regression (LR) [41], Multilayer Perceptron (MLP) [42], Support Vector Machine (SVM) [43], and Extreme Gradient Boosting (XGBoost) [44]. The final model is the meta-learner, demonstrating the best performance. Algorithm 2 illustrates the meta-learner selection process.
**Algorithm 2** Meta-Learner Selection  1:**Initialize** best_performance =0  2:**Initialize** selected_meta_learner =None  3:**for** each classifier in [LR, MLP, SVM, XGBoost] **do**  4:   Train classifier using Stack_models_arr  5:   Evaluate classifier’s performance using experimental analysis  6:   **if** classifier’s performance is better than best_performance **then**  7:     **Update** best_performance with classifier’s performance  8:     **Update** selected_meta_learner with current classifier  9:   **end if**10:**end for**

## 5. Experimental Results

This section presents the results obtained from the DL and meta-learner models and elucidates the dataset and evaluation criteria employed to evaluate the proposed approach.

### 5.1. Dataset

IoT-Botnet 2020 dataset [45] is utilized to evaluate the proposed approach. This CSV-formatted dataset was created by analyzing the BoT-IoT dataset [46] PCAP files. It includes a more interesting collection of streaming and network features. Attacks such as denial of service, distributed denial of service, reconnaissance, and information theft are all included in the IoT-Botnet 2020 dataset. Table 4 outlines Dataset’s records distribution.

The 625,783 records of the IoT-Botnet 2020 dataset are distributed into 8 main attacks as Tabulated in Table 5.

Meta-learning aims to improve a model’s generalizability and adaptability by training it on several datasets. Consequently, the IoT-Botnet 2020 Dataset has been divided into three distinct datasets. Dataset 1 comprises instances of Mirai-UDP Flooding, Dataset 2 consists of Mirai-Hostbruteforce, and Dataset 3 encompasses Mirai-HTTP Flooding. Furthermore, each dataset has a set of normal traffic records (40,073). The Pareto’s 80/20 rule [47] is employed to partition the dataset. This involves dividing the dataset into training and testing segments, with 80% allocated for training and 20% for testing purposes. Table 6 shows each dataset’s records distribution.

### 5.2. Evaluation Metrics

We measure the efficacy of the proposed approach for identifying IoT attacks by employing diverse evaluation metrics. This encompasses Accuracy, F1-Measure, False-Positive Rate (FPR), Recall, and Precision. The subsequent formulas are applied to compute these metrics:

(8) $$\mathrm{Precision}=\frac{\mathrm{TP}}{\mathrm{TP}+\mathrm{FP}}$$

(9) $$\mathrm{Recall}=\mathrm{Detection}\;\mathrm{Rate}=\frac{\mathrm{TP}}{\mathrm{TP}+\mathrm{FN}}$$

(10) $$\mathrm{False}\;\mathrm{Postive}\;\mathrm{Rate}=\frac{\mathrm{FP}}{\mathrm{TN}+\mathrm{FP}}$$

(11) $$\mathrm{True}\;\mathrm{Negative}\;\mathrm{Rate}=\frac{\mathrm{TN}}{\mathrm{TN}+\mathrm{FP}}$$

(12) $$\mathrm{Accuracy}=\frac{\mathrm{TP}+\mathrm{TN}}{\mathrm{TP}+\mathrm{TN}+\mathrm{FP}+\mathrm{FN}}$$

(13) $$F1\;\mathrm{Measure}=2\times (\frac{\mathrm{Precision}\times \mathrm{Recall}}{\mathrm{Precision}+\mathrm{Recall}})$$

The above-mentioned evaluation metrics are generally accepted as standard measures for evaluating the efficacy of IDS. Furthermore, these metrics have been extensively used in prior works such as [3,48,49,50].

### 5.3. The Performance of DL Models

**Figure 2:**
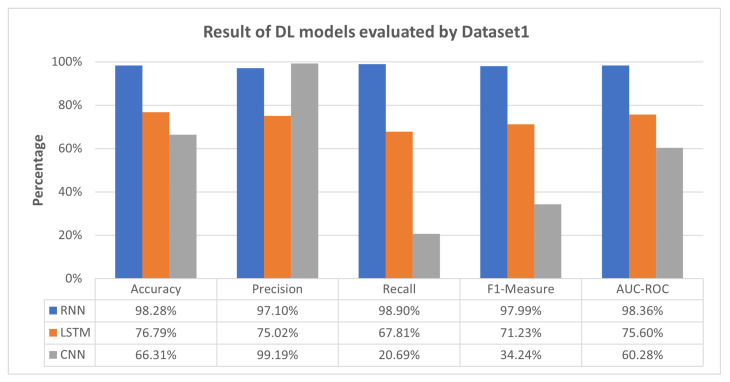
Result of DL models evaluated by Dataset 1.

**Figure 3:**
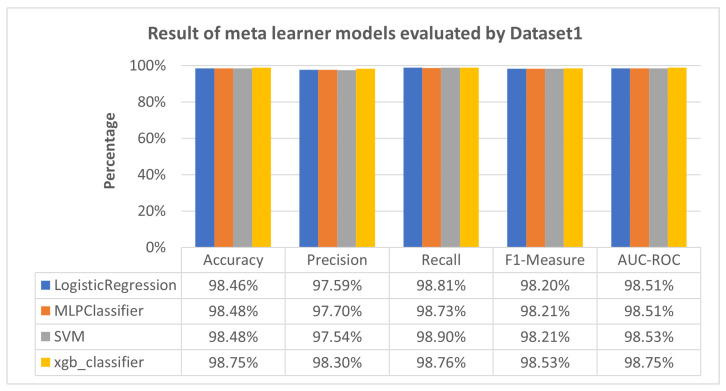
Result of meta-learner models evaluated by Dataset 1.

This section elaborates on the results obtained through implementing deep-learning models within the scope of this study. The evaluation process encompassed three distinct scenarios: Scenario 1, we trained the RRN on Dataset 1 and subsequently assessed its performance across Dataset 1, Dataset 2, and Dataset 3. Scenario 2, the LSTM model underwent training on Dataset 2, following which its efficacy was evaluated across Dataset 1, Dataset 2, and Dataset 3. Scenario 3, the CNN was trained using Dataset 3 and was evaluated across Dataset 1, Dataset 2, and Dataset 3. We employed the Stack_models_arr in each scenario to train multiple meta-learner models (LR, MLP, SVM, and XGBoost). These models were then rigorously evaluated across Dataset 1, Dataset 2, and Dataset 3 to comprehensively assess their capacity for adaptable and generalized performance. The parameters employed in the DL models correspond to those utilized in [37]. We utilized the default parameters for the meta-learner models. The outcome of Scenario 1 is illustrated in Figure 2 and Figure 3.

As shown in Figure 2, RNN achieved a notable accuracy of 98.28%, showcasing its strong predictive abilities. The LSTM model yielded an accuracy of 76.79%, demonstrating decent classification performance. Meanwhile, the CNN achieved an accuracy of 66.31%, indicating competent but comparatively lower predictive power. As for precision, RNN showcased high precision at 97.10%, implying fewer false positives. The LSTM maintained a precision of 75.02%, indicating moderate precision with reduced false positives. Strikingly, the CNN exhibited remarkably high precision at 99.19%, resulting in minimal false positives. Recall rates revealed varied capacities for identifying positive instances. The RNN exhibited robust recall at 98.90%, the LSTM showed a moderate recall of 67.81%, and the CNN struggled with a recall of 20.69%, indicating its difficulty in identifying positive cases. RNN achieved a high F1-measure of 97.99%, while the LSTM’s F1-measure settled at 71.23% and the CNN’s at 34.24%.As for AUC-ROC scores, RNN’s strong distinction with an AUC-ROC of 98.36%, the LSTM’s satisfactory discrimination at 75.60%, and the CNN’s at 60.28%.

**Figure 4:**
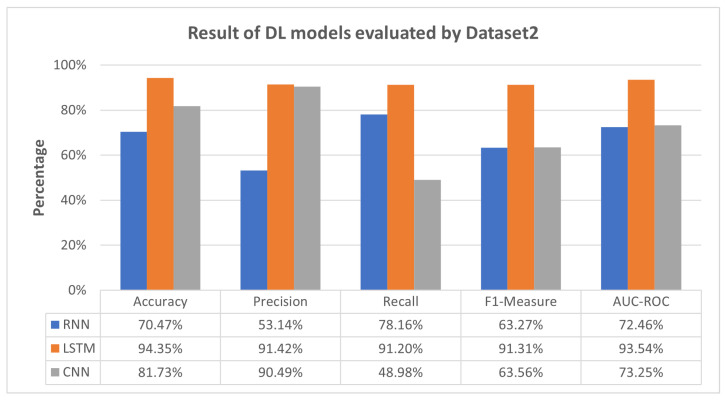
Result of DL models evaluated by Dataset 2.

**Figure 5:**
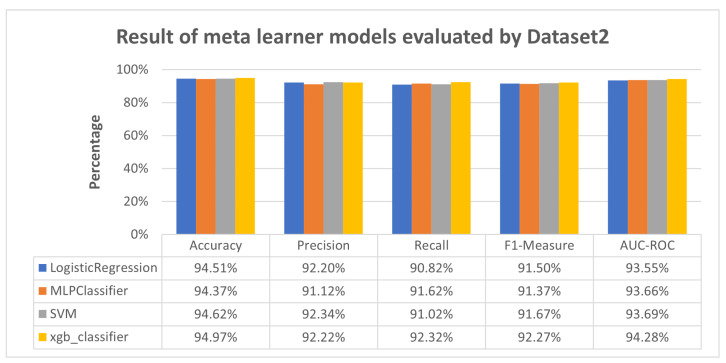
Result of meta-learner models evaluated by Dataset 2.

Figure 3 shows that LR, MLP, SVM, and XGBoost models demonstrated accuracy levels of 98.46%, 98.48%, 98.48%, and 98.75%, respectively. These models consistently exhibited strong precision, recall, F1-measure, and AUC-ROC scores, emphasizing their robustness in discerning between positive and negative instances in IoT attack detection scenarios. These findings contribute valuable insights to enhance the practical application of these models in similar contexts of IoT attack detection. The outcome of Scenario 2 is illustrated in Figure 4 and Figure 5.

Figure 4 reveals that the LSTM model achieved consistently high performance across metrics, with a notable balance between precision and recall. The RNN displayed moderate performance with room for improvement in precision, while the CNN exhibited strengths in precision but faced challenges in recall. These insights emphasize the models’ distinct attributes and performance characteristics when dealing with Dataset 2.

**Figure 6:**
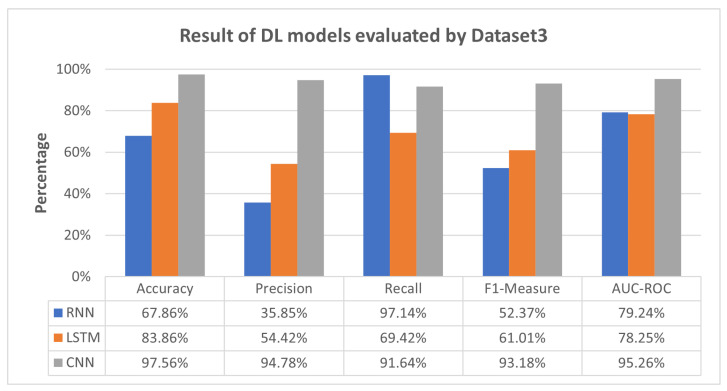
Result of DL models evaluated by Dataset 3.

**Figure 7:**
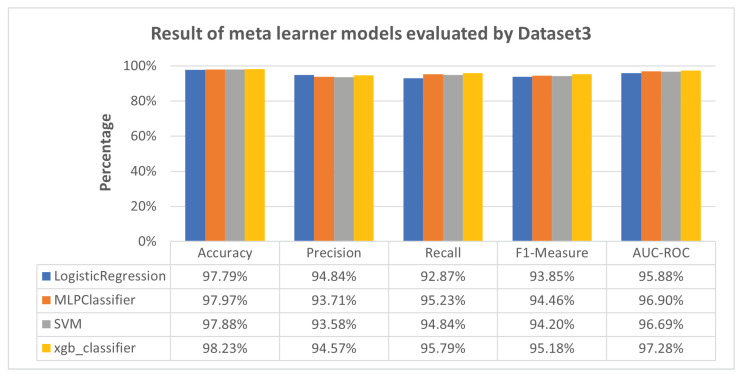
Result of meta-learner models evaluated by Dataset 3.

Figure 5 reveals that the meta-learner models across multiple algorithms consistently showcased high accuracy and balanced performance in precision, recall, F1-measure, and AUC-ROC when evaluated using Dataset 2. The outcome of Scenario 3 is illustrated in Figure 6 and Figure 7.

Figure 6 shows that the CNN model excelled with consistently high performance across metrics, showcasing remarkable precision and recall. The LSTM model exhibited a commendable equilibrium between precision and recall, while the RNN model exhibited challenges in precision but excelled in recall. These insights delineate the models’ unique characteristics and performance dynamics when confronted with Dataset 3.

Figure 7 the meta-learner models, across various algorithms, consistently showcased high accuracy and balanced performance in the precision, recall, F1-measure, and AUC-ROC when evaluated using Dataset 3. These results underscore the effectiveness of these models in handling unseen datasets.

### 5.4. Discussion

The proposed approach demonstrates the capability of the intended meta-learner to detect IoT-based attacks across diverse testing scenarios effectively. In Figure 2, it is evident that LSTM and CNN exhibit moderate performance across all evaluation metrics, while RNN consistently achieves high performance. This performance disparity can be attributed to RNN being exclusively trained and tested using Dataset 1. Conversely, LSTM and CNN were trained on Dataset 2 and Dataset 3, respectively, and subsequently tested on Dataset 1, which was treated as unseen data for LSTM and CNN. This variance underscores the limitations of LSTM and CNN in detecting previously unseen attacks, such as Mirai-UDP Flooding. Addressing the challenge of detecting unseen data is a central focus of our proposed meta-learner approach, as demonstrated in Figure 3.

Figure 3 reveals that all meta-learner models, including LR, MLP, SVM, and XGBoost, outperform LSTM and CNN, exhibiting a substantial enhancement and, in some instances, slightly surpassing the performance of the RNN model. This significant improvement using meta-learner models underscores their potential to handle the challenges posed by previously unseen attack patterns. The meta-learner’s strength lies in its ability to leverage insights from multiple models and datasets, transcending the constraints of single-model approaches. By assimilating knowledge from a broader spectrum of information sources, meta-learners become adept at recognizing intricate relationships, variations, and generalizable features that may elude individual models. This empowers the meta-learner to identify better-nuanced attack patterns, even those not explicitly encountered during its training phase.

Meanwhile, Figure 4 presents a similar pattern, where RNN and CNN exhibit moderate performance across all evaluation metrics, while LSTM achieves high performance. The performance variance can be attributed to the fact that LSTM was exclusively trained and tested using Dataset 2. In contrast, RNN and CNN were trained on Dataset 1 and Dataset 3, respectively, and subsequently tested on Dataset 2, which was treated as unseen data for RNN and CNN. This again highlights RNN and CNN’s limitations in detecting previously unseen attacks, such as Mirai-Hostbruteforce. However, as depicted in Figure 5, the proposed meta-learner approach effectively mitigates these limitations by consistently outperforming RNN and CNN, even surpassing the performance of LSTM in some aspects.

Furthermore, Figure 6 shows RNN and LSTM performing moderately across all evaluation metrics (accuracy, precision, recall, F1-measure, and AUC-ROC), while CNN excels. This variation can be attributed to the fact that CNN was exclusively trained and tested using Dataset 3. On the other hand, RNN and LSTM were trained on Dataset 1 and Dataset 2, respectively, and subsequently tested on Dataset 3, treated as unseen data for RNN and LSTM. This discrepancy underscores the limitations of RNN and LSTM in detecting previously unseen attacks, such as Mirai-HTTP Flooding. Nevertheless, the proposed meta-learner approach, exemplified in Figure 7, once again proves its effectiveness by consistently outperforming RNN and LSTM, exhibiting a substantial improvement and sometimes surpassing the performance of the CNN model.

The consistently superior performance of the RNN model across all testing scenarios is notable, achieving the highest levels of accuracy (98.28%), recall (98.90%), F1-measure (97.99%), and AUC-ROC (98.36%). Notably, the CNN model excels in precision, boasting an impressive 99.19%. However, among the meta-learner models, XGBoost emerges as the leader, showcasing the highest accuracy (98.75%), precision (98.30%), F1-measure (98.53%), and AUC-ROC (98.75%). Meanwhile, the SVM model achieves the highest recall (98.90%), showcasing a marginal improvement of 0.14% over XGBoost in this specific metric.

The proposed approach effectively detects IoT-based attacks across diverse testing scenarios. In Figure 2, we note that RNN consistently outperforms LSTM and CNN. This disparity arises because RNN exclusively uses Dataset 1, while LSTM and CNN were trained on Dataset 2 and Dataset 3, respectively, and tested on Dataset 1 as unseen data. This highlights LSTM and CNN’s limitations in detecting previously unseen attacks, which our meta-learner approach addresses (Figure 3).

Figure 3 demonstrates that all meta-learner models, including LR, MLP, SVM, and XGBoost, outperform LSTM and CNN. Meta-learners leverage insights from multiple models and datasets, enhancing their ability to handle challenges posed by previously unseen attack patterns.

In Figure 4, LSTM excels, having been exclusively trained and tested using Dataset 2, while RNN and CNN, trained on Dataset 1 and Dataset 3, respectively, show moderate performance. Figure 5 confirms the superiority of our meta-learner approach over LSTM, RNN, and CNN.

Figure 6 reveals CNN’s excellence, solely trained and tested using Dataset 3, while RNN and LSTM, trained on Dataset 1 and Dataset 2, exhibit moderate performance. Figure 7 reinforces the effectiveness of our meta-learner approach over individual models.

RNN consistently achieves high performance, with the highest accuracy (98.28%), recall (98.90%), F1-measure (97.99%), and AUC-ROC (98.36%). Among meta-learner models, XGBoost leads with the highest accuracy (98.75%), precision (98.30%), F1-measure (98.53%), and AUC-ROC (98.75%). The SVM model achieves the highest recall (98.90%), slightly surpassing XGBoost in this metric.

Additionally, we have compared our proposed approach and the approach introduced in [15] as shown in Table 7. To evaluate the effectiveness of the deep-learning model introduced in [15] in detecting unseen attacks, we employed Dataset1 for training the model proposed in [15], while we employed Dataset2 and Dataset3 to be considered as unseen attack scenarios.

In Table 7, we can observe that for Dataset 2, the DNN achieved an accuracy of 85.47%. In contrast, our proposed metalearner, using various classifiers such as Logistic Regression, MLP Classifier, SVM, and xgb_classifier, consistently outperformed with accuracies ranging from 94.37% to 94.97%. Notably, the xgb_classifier achieved the highest accuracy of 94.97%. Similar improvements are observed in the precision, recall, F1Measure, and AUCROC metrics, reinforcing the effectiveness of our meta-learner approach.

In the case of Dataset 3, the DNN exhibited an accuracy of 72.78%, while our proposed metalearner methods, again across various classifiers, achieved significantly higher accuracies ranging from 97.79% to 98.23%. The xgb_classifier stood out with the highest accuracy of 98.23%. These results reflect substantial enhancements in detection accuracy, precision, recall, F1Measure, and AUCROC when applying our meta-learner approach compared to the baseline DNN.

The consistent superiority of our meta-learner approach in detecting unseen IoT attacks can be attributed to its ability to leverage the collective strength of various deep-learning classifiers, generalize effectively, and make informed decisions that minimize misclassifications. This suggests its potential as a robust and adaptable solution for bolstering IoT security.

Furthermore, from a practical standpoint, the implications of achieving higher accuracy in IoT unseen attack detection are substantial. This enhanced accuracy translates to improved threat detection and fewer false positives, ultimately bolstering the security of IoT networks.

Furthermore, we have compared the proposed approach with other approaches, as illustrated in Table 8, including approaches in [16,17,19]. This comparison evaluates these approaches’ capabilities in detecting attacks previously encountered (e.g., attacks included in the training process).

As outlined in Table 8, our proposed approach exhibits notably higher accuracy compared to several existing methods, including those in [19], the Catboost-based approach [16], and the CNN model presented in [17]. However, it is worth noting that the GRU model introduced by Kirubavathi et al. [17] achieved a marginally higher accuracy than our proposed approach in this context. This result can be attributed to a fundamental difference in our methodologies and the usage of default hyperparameters for meta-learners with fine-tuning. However, our approach offers unique advantages in terms of generalization and adaptability.

Our approach leverages ensemble learning, specifically employing a stacking technique to harness the collective strengths of multiple models. Stacking proves particularly effective in mitigating the risk of overfitting specific datasets or model architectures, a common challenge encountered in deep-learning approaches. By amalgamating predictions from diverse models, our approach achieves greater robustness and an enhanced capacity to handle a wide spectrum of attack scenarios, particularly in detecting previously unseen attacks. Stacking capitalizes on the complementary strengths of individual models, therefore fortifying our system’s resilience and adaptability for IoT network attack detection.

## 6. Conclusions and Future Works

This paper introduced a meta-learner approach that aimed to detect IoT attacks. Meta-learner model is constructed based on stacking the output of three DL models: RNN, LSTM, and CNN. Furthermore, the selection of meta-learners is based on the experimental analysis of various models: LR, MLP, SVM, and XGBoost. The proposed approach underwent evaluation utilizing the BoT-IoT 2020 dataset across three distinct scenarios. XGBoost model showcased outstanding performance, achieving high accuracy (98.75%), precision (98.30%), F1-measure (98.53%), and AUC-ROC (98.75%). On the other hand, the SVM model exhibited the highest recall (98.90%), marking a slight improvement of 0.14% over the performance achieved by XGBoost. For future works, we intend to investigate more advanced techniques for constructing the meta-learner model that could lead to improved performance. Exploring different ensemble methods, such as boosting or bagging, could offer insights into creating even more effective meta-learners. Moreover, we intend to delve into transfer learning methodologies, which involve leveraging insights acquired from one IoT environment to enhance detection performance in another context. Finally, we intend to conduct a thorough time complexity and computational analysis of our proposed approach.

## Figures and Tables

**Table 1:** Summary of Studies on IoT-Attacks Detection.

Ref.	Approach and Methods	Algorithms Used	Dataset(s)	Key Findings
[4]	Multiple feature fusion, uniform stacking ensemble, Random Forest meta-classifier	Random Forest (RF)	Network traffic data	Ensemble approach for anomaly detection in network traffic.
[5]	Adaptive ensemble with decision tree, Random Forest, k-nearest neighbors, deep neural network	Decision Tree, Random Forest, k-nearest neighbors (kNN), Deep Neural Network (DNN)	NSL-KDD Test+ dataset	Ensemble for intrusion detection using majority voting and weighted decisions.
[6]	Heterogeneous ensemble with CFS-BA hierarchical feature extraction algorithm	Forest Penalizing Attributes, C4.5, Random Forest (RF)	Not specified	Ensemble with feature enhancement and average voting for base classifiers.
[7]	Meta-learning framework for algorithm selection	Meta-learning for algorithm selection	NSL-KDD dataset	Framework for algorithm selection for intrusion detection.
[8]	Stacked ensemble with Dynamic Feature Selector, various algorithms including CNN + LSTM, GRU, DT, RF	Dynamic Feature Selector (DFS), CNN + LSTM, Bi-directional LSTM (BiLSTM), GRU, Decision Tree (DT), Random Forest (RF)	NSL-KDD, UNSWNB15 datasets	Feature selection and ensemble approach with significant accuracy improvements.
[9]	Stacked ensemble with Naive Bayes, Decision Tree, meta-learning algorithms (MDT, MLR, MMT)	Naive Bayes (NB), Decision Tree (DT), Meta-learning algorithms (MDT, MLR, MMT)	UNSWNB15 dataset	Meta-learning ensemble outperforms base models for intrusion detection.
[10]	FC-net, meta-learning framework for intrusion detection based on deep neural networks	Deep Neural Networks (DNNs)	Not specified	Meta-learning framework with versatile intrusion detection capabilities.
[11]	LS-PIO ensemble for IoT security with local search algorithm	Local Search (LS) algorithm, Multiple one-class classifiers	BoT-IoT, UNSW-NB15, NSL-KDD, and 99 datasets	Ensemble with LS-PIO method surpasses other NIDS methods.
[12]	IoT intrusion detection framework with feature selection, RNNs (SimpleRNN, Bi-LSTM)	Recurrent Neural Networks (RNNs): SimpleRNN, Bi-directional Long Short-Term Memory (LSTM)	BoT-IoT dataset	Feature selection reduces data size while maintaining high recall rates.
[13]	Deep machine-learning algorithms for DoS attack detection using RF, CNN, MLP	Random Forest (RF), Convolutional Neural Network (CNN), Multilayer Perceptron (MLP)	Hash chains and IoT datasets	Deep-learning models for DoS attack detection.
[14]	CNN-based anomaly-based IDS for IoT with efficient IoT traffic examination	Convolutional Neural Network (CNN)	NID Dataset and BoT-IoT datasets	CNN-based model for IoT IDS with high accuracy rates.
[15]	MI-based anomaly detection using deep neural networks (DNNs), compared to CNN, RNN, GRU, LSTM	Deep Neural Networks (DNNs), Convolutional Neural Networks (CNN), Recurrent Neural Networks (RNNs): GRU, LSTM	IoT-Botnet 2020 dataset	DNN-based NIDS model outperforms other deep-learning models in accuracy and FAR reduction.
[16]	Enhanced DBO optimization of Catboost parameters for IoT-Botnet detection	Catboost	Real-world IoT traffic datasets	Optimized Catboost parameters outperform other models in accuracy and F1 score.
[17]	Machine-learning and deep-learning models with feature engineering for Botnet detection	Machine Learning: Support Vector Machine (SVM), Decision Tree, Random Forest, Naive Bayes; Deep Learning: Convolutional Neural Network (CNN), Gated Recurrent Unit (GRU)	IoT-23 dataset	Machine learning (SVM, Decision Tree, Random Forest) and deep-learning (CNN, GRU) models for Botnet detection.
[18]	Collaborative machine-learning model with multiple features for early IoT-Botnet detection	Not specified	IoT-Botnet-specific features	Collaborative model for early IoT-Botnet detection with high accuracy.
[19]	Deep learning for cyber security intrusion detection	RNN, DNNs, restricted Boltzmann machines, deep belief networks, CNNs	BoT-IoT	Deep-learning models for IoT-Botnet detection with high accuracy.

**Table 2:** IoT-based attacks.

Attack Type	Description
Device spoofing	Malicious actors impersonate legitimate IoT devices to gain unauthorized access to networks or services. This can lead to data breaches and unauthorized control.
Firmware exploitation	Attackers target vulnerabilities in IoT device firmware, exploiting them to compromise device functionality or gain unauthorized access.
Data manipulation	Tampering with data transmitted between IoT devices leads to the dissemination of false information or unauthorized control. This can disrupt operations and compromise data integrity.
Insider threats	Malicious actions by individuals with legitimate access to IoT devices or networks. Detecting and mitigating insider threats is crucial for IoT security.
Physical attacks	Involves theft, tampering, or physical damage to IoT devices, leading to security breaches. These attacks can compromise the physical integrity of IoT systems.
IoT-Botnet	Botnets of compromised IoT devices are used for various malicious purposes, including DDoS attacks, spam, and malware distribution. IoT botnets exploit vulnerabilities in IoT devices, posing a significant challenge to security.

**Table 3:** The most prominent security challenges in IoT [25].

Challenge	Description
Data Volume	The extensive use of IoT applications such as smart cities and grids generates vast amounts of sensitive data, which in turn exposes these systems to various security risks and vulnerabilities.
Privacy Protection	IoT nodes contain sensitive data that must be safeguarded against identification and traceability. Privacy is a top concern as enterprises continuously process and use data via IoT devices.
Resource Limitations	IoT devices often come with limited computational power and memory resources, which presents a significant challenge when it comes to implementing and maintaining standard security protocols.
Scalability	The IoT system involves numerous entities, requiring scalable confidentiality and security measures across the network.
Heterogeneity	IoT connects diverse devices with varying complexities, capabilities, and technical specifications. Protocols must support these differences to ensure connectivity.
Interoperability	It’s crucial for security procedures within IoT systems to be designed in a way that does not compromise the operational capabilities of IoT nodes. Inadequate interoperability between security measures and IoT devices can result in technical problems and ultimately lead to user dissatisfaction.
Autonomous control	IoT networks should autonomously configure settings in end devices, eliminating the need for user intervention.
Attack Resistance	IoT end devices are often small and lack physical protection, making them vulnerable to natural disasters and sensor damage.

**Table 4:** Distribution of Dataset Records.

Category	Value
Total count of normal rows	40,073
Total count of attack rows	585,710
Total count of rows (normal+attack)	625,783
Total count of features	85

**Table 5:** Attacks distribution in IoT-Botnet 2020 Dataset.

Attack Name	Number of Records
Mirai-UDP Flooding	183,554
Mirai-Hostbruteforceg	121,181
DoS-Synflooding	59,391
Mirai-HTTP Flooding	55,818
Mirai-Ackflooding	55,124
Scan Port OS	53,073
MITM ARP Spoofing	35,377
Scan Hostport	22,192

**Table 6:** Records distribution of each dataset.

Dataset	Training Dataset (80%)	Testing Dataset (20%)
Dataset 1 Mirai-UDP Flooding	178,901	44,725
Dataset 2 Mirai-Hostbruteforceg	129,002	32,251
Dataset 3 Mirai-HTTP Flooding	76,664	19,196

**Table 7:** Comparison between the proposed approach and the approach in [15].

Testing Dataset	Algorithm		Accuracy	Precision	Recall	F1-Measure	AUC-ROC
Dataset 2	DNN [15]		85.47%	90.43%	90.22%	90.22%	80.67%
	Proposed meta-learner	LR	94.51%	92.20%	90.82%	91.50%	93.55%
		MLP	94.37%	91.12%	91.62%	91.37%	93.66%
		SVM	94.62%	92.34%	91.02%	91.67%	93.69%
		XGB	94.97%	92.22%	92.32%	92.27%	94.28%
Dataset 3	DNN [15]		72.78%	69.93%	93.38%	79.97%	68.73%
	Proposed meta-learner	LR	97.79%	94.84%	92.87%	93.85%	95.88%
		MLP	97.97%	93.71%	95.23%	94.46%	96.90%
		SVM	97.88%	93.58%	94.84%	94.20%	96.69%
		XGB	98.23%	94.57%	95.79%	95.18%	97.28%

**Table 8:** Comparison between the proposed approach and other approaches.

Dataset	Classifier	Accuracy
Work in [19]		
BoT-IoT [46]	DNN	96.611%
	RNN	96.877%
	CNN	96.919%
Work in [16]		
BoT-IoT [46]	IDBO-Catboost	98.62%
Botnet [51]	IDBO-Catboost	96.10%
Work in [17]		
IoT-23 [52]	CNN	96.69%
	GRU	99.87%
Proposed work		
IoT-Botnet 2020 [45]	LogisticRegression	98.46%
	MLPClassifier	98.48%
	SVM	98.48%
	xgb_classifier	98.75%

## Data Availability

Not applicable.
